# Effects of Exercise Training Combined with Increased Physical Activity to Prevent Chronic Pain in Community-Dwelling Older Adults: A Preliminary Randomized Controlled Trial

**DOI:** 10.1155/2018/2132039

**Published:** 2018-04-05

**Authors:** Tatsuya Hirase, Hideki Kataoka, Shigeru Inokuchi, Jiro Nakano, Junya Sakamoto, Minoru Okita

**Affiliations:** ^1^Department of Physical Therapy Science, Nagasaki University Graduate School of Biomedical Sciences, 1-7-1 Sakamoto, Nagasaki 852-8520, Japan; ^2^Department of Locomotive Rehabilitation Science, Nagasaki University Graduate School of Biomedical Sciences, 1-7-1 Sakamoto, Nagasaki 852-8520, Japan; ^3^Department of Rehabilitation, Nagasaki Memorial Hospital, 11-54 Fukahori, Nagasaki 851-0301, Japan

## Abstract

**Objective:**

With the aim of developing a chronic pain prevention program, this randomized controlled trial examined whether exercise training combined with increased physical activity more effectively improves pain and physical activity than exercise training alone in community-dwelling older adults without chronic pain.

**Methods:**

We randomized 76 older adults without chronic pain into an intervention group (*n*=38) involving exercise training combined with increased physical activity and a control group (*n*=38) involving exercise training alone. The exercise training comprised weekly 60-min sessions for 12 weeks. The program to increase physical activity required participants to record their daily step counts using pedometers. Pain intensity, total number of pain sites, and physical activity were assessed before and 12 weeks after the intervention.

**Results:**

A time-by-group interaction was found for physical activity, with the intervention group showing significant improvement (*p* < 0.05). The intervention group also showed greater improvement in pain intensity and total number of pain sites at 12 weeks after intervention than the control group (*p* < 0.05).

**Conclusions:**

In older adults without chronic pain, exercise training combind with increased physical activity improves key outcome indicators more effectively than exercise training alone. “This trial is registered with UMIN000018503.”

## 1. Introduction

Chronic pain causes increased health-care costs as well as deterioration in quality of life and is common among community-dwelling older adults [1]. Our previous study revealed that 54.4% of community-dwelling older adults had chronic pain, associated with declining physical function, poor psychological status, and low physical activity levels [2]. Kaiho et al. [3] reported that chronic pain is a risk factor for the need for long-term care. Therefore, an effective approach to the prevention of chronic pain in community-dwelling older adults needs to be developed urgently.

In Japan, the number of community-dwelling older adults who require long-term care has increased because of the rapidly aging population. In addition, the average healthy life expectancy is approximately 10 years lower than the mean life expectancy [4]. In 2006, the Japanese Government revised its long-term care insurance system, which provides client-centered services to older adults who are certified as requiring support or care according to their disability levels and introduced new preventive care services to extend the healthy life expectancy of its residents [5]. These care services involve a community-based prevention program to delay the need for long-term care, with exercise classes often provided to older adults assessed as being at risk for requiring long-term care in the near future. Such classes have effectively improved physical function [6], psychological status [7], and physical activity levels [8]. However, in Japan, full assessments of pain, including pain intensity and duration, are not routinely performed during exercise classes because of a lack of recognition that chronic pain is a serious social issue that results in higher health care costs and a declining quality of life [9, 10]. Therefore, the nature of an effective intervention focused on preventing chronic pain in older adults who participate in these classes remains unclear. The development of a community-based intervention program aimed at preventing increased pain intensity in older adults without chronic pain is needed to extend healthy life expectancy in Japan, as the number of older adults with pain is projected to increase over the next 50 years [11].

Park and Hughes [12] reviewed empirical evidence and revealed that exercise training is an effective nonpharmacological approach for managing chronic pain in community-dwelling older adults. In a previous study that used an animal model, Sluka et al. [13] reported that physical inactivity is a risk factor for the development of chronic pain, and that regular physical activity prevents chronic pain. Our previous study revealed that a psychosocial intervention that involved self-management education and increased physical activity, combined with exercise training, more effectively decreased pain intensity than exercise training alone in older adults with chronic pain who participated in community-based exercise classes [14]. Therefore, an intervention program to increase physical activity, combined with exercise training, may be beneficial to preventing chronic pain in older adults.

On the basis of the findings of previous studies [12–14], we hypothesized that a community-based intervention to increase physical activity combined with exercise training, with the aim of developing of a chronic pain prevention program, would more effectively decrease pain intensity and improve physical activity in older adults without chronic pain than exercise training without increased physical activity. The aim of this study was to investigate that hypothesis.

## 2. Materials and Methods

### 2.1. Participants

We enrolled older adults participating in community exercise classes once a week in the Japanese city of Unzen, selecting seven exercise classes supported by physical therapists. Physical therapists were asked to choose potential participants aged ≥65 years who were living at home, able to walk outdoors without a cane, and able to independently perform activities of daily living. We excluded older adults who had chronic pain, defined as the presence of related symptoms within the past month that continued for at least 6 months and corresponded to a Numerical Rating Scale (NRS) of at five or more at the maximum pain site [9]. We also excluded older adults who had exercised at least four times during the month before the initial interview and those who had musculoskeletal, neurological, or cardiovascular conditions that may be aggravated by exercise, according to the judgments of their primary care doctors. Moreover, we excluded older adults who were unable to respond to interview questions because of cognitive impairment.

Each participant provided written informed consent in accordance with the guidelines of the Nagasaki University Graduate School of Medicine and the 2008 Helsinki Declaration of Human Rights. The Ethics Review Board of Nagasaki University Graduate School of Biomedical Sciences approved this study.

### 2.2. Design and Randomization

This randomized controlled trial was performed from August 2015 to November 2016. Participants who met the inclusion criteria were randomized into two groups (1 : 1) using computer-generated randomization lists. The groups comprised an intervention group that involved increased physical activity combined with exercise training and a control group that involved exercise training alone. An independent investigator performed the randomization after baseline assessment. Physical therapists who supported the community exercise classes assessed the participants and implemented the intervention program.

### 2.3. Interventions

We asked participants in both groups to attend a 60 min weekly exercise class for 12 weeks. The class comprised 10 min of warm-up, 20 min of strength training, 20 min of balance training, and 10 min of cool-down, as described in our previous study [14]. All classes involved approximately 10 participants. The intensity of the strength and balance training during the 40 min was constant over the course of the intervention period and included a total 10 min of breaks, depending on the participants' physical capacity. As a participant resource, and to ensure the consistency of the training, we provided a videotape of the correct executions of the exercises, and instructors were supervised by the physical therapists who supported each class.

The intervention group also participated in a program to increase physical activity during the 12-week intervention period. Only participants in this group were given pedometers (Yamax Digiwalker SW-200; Yamasa Tokei Keiki Co., Ltd., Tokyo, Japan), which they wore at all times they were awake, and daily diaries to record their daily step counts. Yamada et al. [15] reported that an intervention followed by goal setting to progressively increase daily step counts, self-monitoring, and feedback is a useful method for increasing the physical activity of community-dwelling older adults. On the basis of these findings, the physical therapists supporting the community exercise classes checked participants' diaries once a week and advised them to increase their daily step counts by approximately 10% relative to their baseline during the first month. During the second and third months, we advised participants in the intervention group to increase their step counts by approximately 20% and 30%, respectively, relative to their baseline. Participants in this group were asked to record their daily step counts at the end of each day; written activity logs were averaged weekly to determine whether the participants were achieving their step goal. Physical therapists checked the participants' daily step counts during their weekly exercise class and provided feedback and encouragement for 10 minutes during the class, so that the participants could achieve their step goal.

### 2.4. Assessment

The primary outcome of this study was pain intensity, and the secondary outcomes were physical function, psychological status, and physical activity level. Before the study began, the physical therapists received training from one of the authors (Tatsuya Hirase) on the assessment protocols.

Pain was assessed by determining the total number of pain sites and intensity at the maximum pain site, using NRS. Participants used a body chart to identify their pain sites and scored their pain intensity according to NRS.

Physical function was assessed using the Chair Stand Test (CST) [16] and the Timed Up-and-Go (TUG) test [17]. These tests were conducted twice, and the best value from the two tests was recorded.

The psychological status was evaluated using the 15-item version of the Geriatric Depression Scale (GDS-15) [18] and the Pain Catastrophizing Scale (PCS) [19]. The PCS includes the same 13 items reported by Sullivan et al. [20] and comprises three categories: rumination (five items), helplessness (five items), and magnification (three items). Each item is assessed on a five-point scale ranging from zero (“not at all”) to four (“all the time”), with a total score ranging from 0 to 52, and higher scores indicating greater catastrophizing. Pain, physical function, and the psychological status in both groups were evaluated before and within 7 days of the end of the 12-week program.

Participants in both groups wore a pedometer with an accelerometer (Kenz Lifecorder GS; Suzuken Co., Ltd., Nagoya, Japan) to assess their physical activity levels during the same 2-week periods—the first week of the study and the final week of the study—to evaluate the preintervention and postintervention effects. Participants were instructed to wear the pedometer on their belt or waistband, above the right midline of the thigh, from the moment they got out of bed in the morning until they went to bed in the evening, except while bathing or swimming. Based on a previous report by Matsubara et al. [21], we calculated the participants' mean daily step counts and activity times for mild (1–3 metabolic equivalents (METS)), moderate (4–6 METS), and heavy (7–9 METS) exercise during each period with the Lifecorder GS (Suzuken Co., Ltd.) pedometer.

### 2.5. Required Sample Size

We designed this study to detect an effect size of 0.61, according to the results of our previous study [14]. With a statistical significance level of 5% (*p* ≤ 0.05), a statistical power of 80%, and allowance for a 5% dropout rate, we required a minimum of 36 participants in each group.

### 2.6. Statistical Analysis

Statistical analyses were performed using SPSS 22.0 for Windows (IBM Corp., Armonk, NY, USA). We used unpaired *t*-tests to evaluate significant differences in age, height, body weight, and outcome measures between the two groups before the intervention, and chi-square tests for group comparisons of sex distribution and proportion of dropouts. We analyzed the effects of the intervention program on the outcome measures using a 2 × 2 (time (baseline and 12-week postintervention) × group (intervention and control groups)) analysis of variance. We used post hoc Bonferroni tests for specific comparisons, and significance was two-sided.

## 3. Results

**Figure 1:**
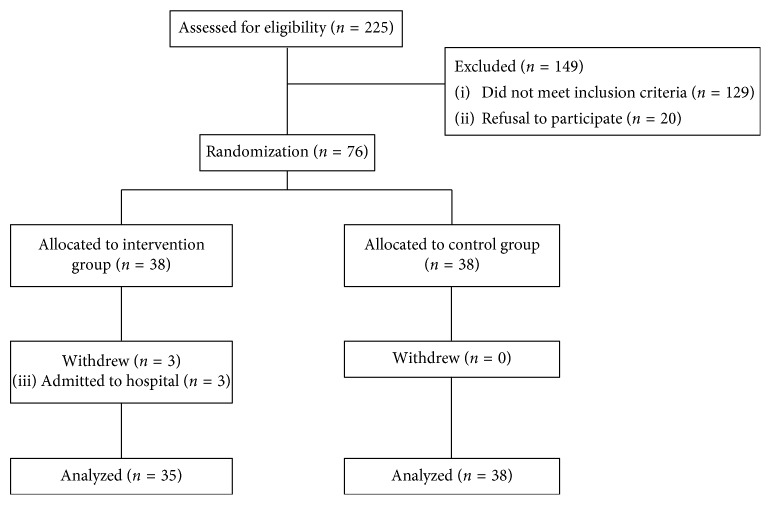
Flowchart outlining the study's participant recruitment process and randomization into the two study groups.

Figure 1, a flow chart, outlines the study's recruitment of participants and randomization into the two study groups. A total of 225 older adults were screened as potential participants; 149 either declined to participate (*n*=20) or failed to meet the inclusion criteria (*n*=129). Of those who did not meet the inclusion criteria, 120 had chronic pain, five were unable to respond to interview questions because of cognitive impairment, and four had exercised four or more times in the month before the initial interview. We enrolled the remaining 76 older adults into the study, and randomly allocated each of them to either the intervention group (*n*=38) or the control group (*n*=38). Three participants in the intervention group (3.9% of the sample) withdrew from the trial; they were admitted to hospital because of serious illness (pneumonia (*n*=2) and heart disease (*n*=1)). There were no significant group differences in study withdrawal (*p*=0.240), and no participants dropped out for reasons relating to the intervention program itself. Seventy-three participants completed the 12-week intervention: 35 in the intervention group and 38 in the control group.

During the intervention period, participants in the intervention and control groups who completed the study attended 91.2% and 90.6% of the classes, respectively. This difference in class attendance was not significant between the groups (*p*=0.817).

### 3.1. Baseline Characteristics

Table 1 summarizes the participants' baseline characteristics. There were no significant differences in age, sex, pain, physical function, psychological status, or physical activity between the two groups (*p* ≥ 0.157 for all comparisons).

### 3.2. Effects of the Interventions on Pain, Physical Function, Psychological Status, and Physical Activity

Table 2 shows the effects of the interventions on the outcome measures in the preintervention and postintervention periods. There was a significant time-by-group interaction for daily step counts (*p*=0.022).

#### 3.2.1. Effects of the Interventions on the Primary Outcome for Both Groups

The mean number of total pain sites and NRS score at the maximum pain site at 12 weeks after intervention in the intervention group were significantly better than the values in the control group (*p*=0.016 and *p*=0.002, resp.). Within the control group, the mean NRS score at the maximum pain site at 12 weeks after intervention was significantly worse than that before intervention (*p*=0.027). We found no significant difference in the preintervention and postintervention mean values within the intervention group.

#### 3.2.2. Effects of the Interventions on the Secondary Outcomes for Both Groups

The mean daily step count in the intervention group increased by 20.0% (from 4312.9 ± 2392.5 to 5175.7 ± 3126.1) and that in the control group decreased by 3.1% (from 4030.2 ± 2266.5 to 3918.6 ± 1870.3). The mean daily step count and moderate activity time at 12 weeks after intervention in the intervention group were significantly better than those in the control group (*p*=0.039 and *p*=0.017, resp.). Within the intervention group, the mean daily step count and mild activity time at 12 weeks after intervention improved significantly compared with the preintervention values (*p*=0.006 and *p*=0.015, resp.). No similar improvement was seen in the control group values.

We found no significant differences in mean CST and TUG scores at 12 weeks after intervention between the two groups. Within both groups, the mean at 12 weeks after intervention had improved significantly compared with the preintervention values (*p* < 0.001).

We found no significant differences in mean values at 12 weeks after intervention for GDS-15, total PCS, rumination, helplessness, and magnification scores between the two groups. There were also no significant within-group differences in the mean values between the preintervention and postintervention time points.

## 4. Discussion

This randomized controlled trial revealed that a community-based intervention program that combined exercise training with increased physical activity effectively improved physical activity and prevented worsening of pain intensity in older adults without chronic pain. In Japan, the front-runner of “super-aged societies,” efforts to extend healthy life expectancy are needed because the number of community-dwelling older adults who require long-term care has increased because of rapid population aging. In addition, the development of an effective intervention to prevent chronic pain is urgently needed because the proportion of older adults with chronic pain is high, and chronic pain is associated with the need for long-term care. Our data suggest that exercise training in combination with increased physical activity is beneficial in preventing chronic pain in older adults who have not yet experienced chronic pain. Consequently, this study is both novel and important, because, to the best of our knowledge, it is the first to test the efficacy of a community-based intervention program for preventing chronic pain in older adults.

Again, to the best of our knowledge, no previous studies have investigated the preventive effects of a community-based intervention program on pain in community-dwelling older adults without chronic pain, as most studies have targeted individuals with chronic pain [1, 12, 14]. In previous animal model studies that used mice, Sluka et al. [13] reported that an 8-week wheel-running activity intervention effectively prevented decreases in muscle withdrawal thresholds and increased responses to mechanical stimulation of the paw for up to 72 hours after the noninflammatory pain model was induced with two injections of unbuffered pH 4.0 saline into one gastrocnemius muscle 5 days apart, compared with sedentary controls. Grace et al. [22] showed that a 6-week voluntary wheel-running intervention with mice before chronic constriction injury prevented the full development of allodynia for up to 3 months duration of the injury. These studies suggest that increased physical activity is beneficial for preventing chronic pain. In the current study, to increase physical activity, participants in the intervention group were given pedometers and daily diaries and asked to record their daily step counts. Additionally, physical therapists who supported the community exercise classes checked participants' diaries once a week and advised them to progressively increase their daily step counts during the 12-week intervention period. As a result, we found a time-by-group interaction for daily step counts, with participants in the intervention group showing significant improvement and moderate activity times 12 weeks after intervention compared with the control group. Moreover, the total number of pain sites and maximum pain site scores improved significantly compared with the control group 12 weeks after intervention in the intervention group. Consequently, our results suggest that exercise training combined with increased physical activity effectively improves physical activity levels, which leads to a relatively low number of total pain sites and decreased pain intensity, ultimately preventing the development of chronic pain in older adults. In the control group, pain scores at the maximum pain site 12 weeks after intervention significantly worsened compared with the equivalent preintervention value, and no significant difference was observed in the preintervention and postintervention within the intervention group. These results may suggest that only exercise intervention once a week is insufficient to decrease pain intensity and to prevent the development of chronic pain; however, exercise training combined with increased physical activity can prevent the development of chronic pain. Although the detailed mechanism is unclear, increasing physical activity would be important in preventing chronic pain, because participants' physical activity levels did not change in the control group, but it increased in the intervention group.

Within both groups, CST and TUG scores, as markers of lower extremity muscle strength [16] and walking ability [17], improved significantly during the 12-week intervention period, and there were no significant differences in the mean values at 12 weeks after intervention between the two groups. Previous studies have reported that exercise programs that involve muscle strength and balance training significantly improve physical function in community-dwelling older adults, according to CST and TUG scores [6, 23]. Our results were similar to those of previous studies and suggested that exercise training leads to improved physical function in community-dwelling older adults without chronic pain.

Regarding the psychological status, there were no significant differences in the mean GDS-15 and PCS values at 12 weeks after intervention between the two groups. Vlaeyen and Linton [24] showed that pain-related catastrophizing is an important factor in the development of chronic pain, which can cause disability, depression, and low physical activity levels. Our previous study [14] revealed that a psychosocial intervention that leads to improved PCS scores effectively decreases pain intensity and improves physical activity levels in older adults with chronic pain. The findings of the abovementioned studies suggest that intervention programs that modify the cognitive aspects of pain are beneficial in older adults suffering from chronic pain. In the present study, the participants' psychological status may not have changed because our study targeted older adults without chronic pain, and as such the provision of an intervention program to modify the cognitive aspects of pain was out of scope.

During this study, no study-related adverse events occurred. The intervention and control groups had similarly low withdrawal rates and high rates of participation in the exercise sessions. These findings suggest that exercise training combined with increased physical activity was widely embraced by the participants and is a safe and feasible intervention during community-based exercise classes.

Our study has several limitations. The first limitation is that the physical therapists who assessed the participants also conducted the intervention programs. Therefore, our results may have been influenced by the physical therapists' expertise and/or reporting bias during the community exercise classes. In saying this, all the physical therapists who participated in the study received the same level of training from a senior physical therapist before the trial began, and we videotaped the exercise program to ensure consistency. Therefore, we believe that the physical therapists' expertise had only minimal effects on the study results. The second limitation is that our 12-week intervention period was relatively short. Therefore, further study is needed to evaluate the long-term benefits of this preliminary randomized controlled trial.

## 5. Conclusions

An intervention program that involved exercise training combined with increased physical activity, with the aim of developing a chronic pain prevention program to extend healthy life expectancy, improved pain and physical activity levels more effectively than exercise training alone in a sample of older adults without chronic pain. The intervention program was widely embraced by these older adults. Therefore, we believe that exercise training combined with increased physical activity is an effective and feasible intervention program to prevent the development of chronic pain in community-dwelling older adults.

## Figures and Tables

**Table 1:** Participants' baseline characteristics.

Characteristics	Intervention group (*n*=38)	Control group (*n*=38)	*p* value
Age (years)	78.3 ± 5.8	78.3 ± 6.5	0.956
Female, *n* (%)	29 (76.3)	29 (76.3)	0.999
Height (cm)	150.7 ± 7.6	152.5 ± 7.0	0.303
Weight (kg)	50.3 ± 9.0	52.6 ± 8.9	0.252
Total number of pain sites	1.7 ± 1.5	2.0 ± 2.0	0.566
NRS at the site of maximum pain	2.4 ± 2.2	2.7 ± 2.4	0.555
CST (s)	7.7 ± 2.7	7.4 ± 2.0	0.544
TUG (s)	7.6 ± 1.6	7.6 ± 1.6	0.938
GDS-15 score (points)	3.2 ± 3.5	2.7 ± 2.9	0.497
PCS total score (points)	23.3 ± 13.1	23.4 ± 11.1	0.963
Rumination score (points)	11.0 ± 6.1	10.8 ± 4.7	0.850
Helplessness score (points)	7.2 ± 4.7	7.6 ± 4.3	0.704
Magnification score (points)	5.1 ± 3.7	5.2 ± 3.3	0.974
Daily step counts (steps)	4533.1 ± 2515.8	4030.2 ± 2266.5	0.363
Mild activity times (s)	2471.7 ± 1368.2	2382.8 ± 1158.3	0.762
Moderate activity times (s)	484.9 ± 683.0	303.0 ± 442.0	0.157
Heavy activity times (s)	17.2 ± 26.0	20.0 ± 49.7	0.761

Values are expressed as mean ± standard deviation (SD). NRS: Numerical Rating Scale; CST: Chair Stand Test; TUG: Timed Up-and-Go test; GDS-15: 15-item version of the Geriatric Depression Scale; PCS: Pain Catastrophizing Scale.

**Table 2:** Group comparisons of outcome measures during the intervention period.

Item	Intervention group (*n*=35)			Control group (*n*=35)			Time-by-group interaction	
	Preintervention	After 12 weeks	Mean difference	Preintervention	After 12 weeks	Mean difference	*F* value	*p* value
Total number of pain sites	1.7 ± 1.4	1.3 ± 1.1^a^	−0.3 ± 1.0	2.0 ± 2.0	2.2 ± 1.6	0.2 ± 1.7	2.622	0.110
NRS at the site of maximum pain	2.3 ± 2.1	2.1 ± 1.6^a^	−0.2 ± 1.5	2.7 ± 2.4	3.6 ± 2.4^b^	0.9 ± 3.2	3.642	0.060
CST (s)	7.7 ± 2.8	7.0 ± 2.5^b^	−0.7 ± 1.4	7.4 ± 2.0	6.7 ± 1.8^b^	−0.7 ± 1.0	0.012	0.913
TUG (s)	7.6 ± 1.7	7.0 ± 1.3^b^	−0.6 ± 1.0	7.6 ± 1.6	7.2 ± 1.7^b^	−0.5 ± 0.7	0.529	0.469
GDS-15 score (points)	3.0 ± 3.3	2.5 ± 3.1	−0.5 ± 2.0	2.7 ± 2.9	2.6 ± 2.4	−0.1 ± 2.3	0.490	0.486
PCS total score (points)	23.0 ± 13.3	20.8 ± 11.5	−2.2 ± 8.1	23.4 ± 11.1	23.6 ± 12.9	0.2 ± 10.2	1.200	0.277
Rumination score (points)	10.7 ± 6.2	9.6 ± 5.4	−1.1 ± 3.8	10.8 ± 4.7	11.3 ± 5.4	0.5 ± 4.9	2.271	0.136
Helplessness score (points)	7.2 ± 4.8	7.0 ± 4.1	−0.2 ± 3.8	7.6 ± 4.3	7.5 ± 4.9	−0.1 ± 4.5	0.005	0.947
Magnification score (points)	4.9 ± 3.6	4.2 ± 2.9	−0.7 ± 2.2	5.2 ± 3.3	4.9 ± 3.2	−0.3 ± 2.5	0.660	0.419
Daily step counts (steps)	4312.9 ± 2392.5	5175.7 ± 3126.1^ab^	862.8 ± 1933.9	4030.2 ± 2266.5	3918.6 ± 1870.3	−111.6 ± 1630.9	5.445	0.022
Mild activity times (s)	2394.2 ± 1399.9	2774.8 ± 1550.8^b^	457.7 ± 1037.3	2382.8 ± 1158.3	2385.0 ± 1049.1	2.2 ± 844.2	3.218	0.077
Moderate activity times (s)	428.1 ± 545.1	555.3 ± 689.8^a^	143.9 ± 422.9	303.0 ± 442.0	255.7 ± 286.0	−47.3 ± 341.4	3.801	0.055
Heavy activity times (s)	16.6 ± 26.9	18.1 ± 29.4	1.6 ± 31.2	20.0 ± 49.7	20.5 ± 40.8	0.5 ± 35.1	0.016	0.900

Values are expressed as mean ± standard deviation (SD). NRS: Numerical Rating Scale; CST: Chair Stand Test; TUG: Timed Up-and-Go test; GDS-15: 15-item version of the Geriatric Depression Scale; PCS: Pain Catastrophizing Scale. ^a^Significant group difference (*p* < 0.05), ^b^Significant preintervention to postintervention difference (*p* < 0.05).

## Data Availability

The dataset analyzed in the current study is available from the corresponding author on reasonable request.

## References

[1] Reid M. C., Eccleston C., Pillemer K. (2015). Management of chronic pain in older adults. *BMJ*.

[2] Hirase T., Kataoka H., Inokuchi S., Nakano J., Sakamoto J., Okita M. (2017). Factors associated with chronic musculoskeletal pain in Japanese community-dwelling older adults: a cross-sectional study. *Medicine*.

[3] Kaiho Y., Sugawara Y., Sugiyama K. (2017). Impact of pain on incident risk of disability in elderly Japanese: cause-specific analysis. *Anesthesiology*.

[4] Tokudome S., Hashimoto S., Igata A. (2016). Life expectancy and healthy life expectancy of Japan: the fastest graying society in the world. *BMC Research Notes*.

[5] Tsutsui T., Muramatsu N. (2007). Japan’s universal long-term care system reform of 2005: containing costs and realizing a vision. *Journal of the American Geriatrics Society*.

[6] Hasegawa M., Yamazaki S., Kimura M., Nakano K., Yasumura S. (2013). Community-based exercise program reduces chronic knee pain in elderly Japanese women at high risk of requiring long-term care: a non-randomized controlled trial. *Geriatrics and Gerontolology International*.

[7] Inokuchi S., Matsusaka N., Hayashi T., Shindo H. (2007). Feasibility and effectiveness of a nurse-led community exercise programme for prevention of falls among frail elderly people: a multi-centre controlled trial. *Journal of Rehabilitation Medicine*.

[8] Nishiguchi S., Yamada M., Tanigawa T. (2015). A 12-week physical and cognitive exercise program can improve cognitive function and neural efficiency in community-dwelling older adults: a randomized controlled trial. *Journal of the American Geriatrics Society*.

[9] Nakamura M., Nishiwaki Y., Ushida T., Toyama Y. (2011). Prevalence and characteristics of chronic musculoskeletal pain in Japan. *Journal of Orthopaedic Science*.

[10] Inoue S., Kobayashi F., Nishihara M. (2015). Chronic pain in the Japanese community-prevalence, characteristics and impact on quality of life. *PLoS One*.

[11] Suka M., Yoshida K. (2009). The national burden of musculoskeletal pain in Japan: projection to the year 2055. *Clinical Journal of Pain*.

[12] Park J., Hughes A. K. (2012). Nonpharmacological approaches to the management of chronic pain in community-dwelling older adults: a review of empirical evidence. *Journal of the American Geriatrics Society*.

[13] Sluka K. A., O’Donnell J. M., Danielson J., Rasmussen L. A. (2013). Regular physical activity prevents development of chronic pain and activation of central neurons. *Journal of Applied Physiology*.

[14] Hirase T., Kataoka H., Nakano J., Inokushi S., Sakamoto J., Okita M. (2018). Effects of a psychosocial intervention programme combined with exercise in community-dwelling older adults with chronic pain: a randomized controlled trial. *European Journal of Pain*.

[15] Yamada M., Nishiguchi S., Fukutani N., Aoyama T., Arai H. (2015). Mail-based intervention for sarcopenia prevention increased anabolic hormone and skeletal muscle mass in community-dwelling Japanese older adults: the INE study. *Journal of the American Medical Directors Association*.

[16] Gardner M. M., Buchner D. M., Robertson M. C., Campbell A. J. (2001). Practical implementation of an exercise-based falls prevention programme. *Age and Ageing*.

[17] Podsiadlo D., Richardson S. (1991). The timed up-and-go: a test of basic functional mobility for frail elderly persons. *Journal of the American Geriatrics Society*.

[18] Sheikh J. I., Yesavage J. A., Brink T. (1986). Geriatric Depression Scale (GDS): recent findings and development of a shorter version. *Clinical Gerontology: A Guide to Assessment and Intervention*.

[19] Iwaki R., Arimura T., Jensen M. P., Hosoi H. (2012). Global catastrophizing vs catastrophizing subdomains: assessment and associations with patient functioning. *Pain Medicine*.

[20] Sullivan M. J., Bishop S. R., Pivik J. (1995). The pain catastrophizing scale: development and validation. *Psychological Assessment*.

[21] Matsubara T., Arai Y. C., Shimo K. (2010). Effects of cognitive-behavioral therapy on pain intensity and level of physical activity in Japanese patients with chronic pain: a preliminary quasi-experimental study. *Journal of Physical Therapy*.

[22] Grace P. M., Fabisiak T. J., Green-Fulgham S. M. (2016). Prior voluntary wheel running attenuates neuropathic pain. *Pain*.

[23] Hirase T., Inokuchi S., Matsusaka N., Okita M. (2015). Effects of a balance training program using a foam rubber pad in community-based older adults. *Journal of Geriatric Physical Therapy*.

[24] Vlaeyen J. W. S., Linton S. J. (2000). Fear-avoidance and its consequences in chronic musculoskeletal pain: a state of the art. *Pain*.

